# Latitudinal effects on crown shape evolution

**DOI:** 10.1002/ece3.4275

**Published:** 2018-07-22

**Authors:** Magnus Lindh, Daniel S. Falster, Lai Zhang, Ulf Dieckmann, Åke Brännström

**Affiliations:** ^1^ Magnus Lindh, Department of Physical Geography Stockholm University Stockholm Sweden; ^2^ Evolution & Ecology Research Centre, and School of Biological, Earth and Environmental Sciences University of New South Wales Sydney NSW Australia; ^3^ School of Mathematical Science Yangzhou University Yangzhou China; ^4^ Evolution and Ecology Program International Institute for Applied Systems Analysis Laxenburg Austria; ^5^ Department of Mathematics and Mathematical Statistics Umeå University Umeå Sweden

**Keywords:** adaptive dynamics, crown, evolution, latitude, productivity, shape, sun angle, tree

## Abstract

Large variations in crown shape are observed across the globe, from plants with wide and deep crowns to those with leaves clustered at the top. While there have been advances in the large‐scale monitoring of forests, little is known about factors driving variations in crown shape with environmental conditions. Previous theoretical research suggests a gradient in crown shape with latitude, due to the effects of sun angle. Yet, it remains unclear whether such changes are also predicted under competition. Using a size‐structured forest‐growth model that incorporates self‐shading from plants and competitive shading from their neighbors, we investigate how changes in site productivity and sun angle shape crown evolution. We consider evolution in two traits describing the top‐heaviness and width‐to‐height ratio of crowns, shaped by trade‐offs reflecting the costs and benefits of alternative architectures. In top‐heavy trees, most of the leaves are at the top half of the trunk. We show that, contrary to common belief, the angle of sun beams per se has only a weak influence on crown shapes, except at low site productivity. By contrast, reduced site productivity has a strong effect, with trees growing in less productive sites keeping their leaves closer to the ground. The crown width‐to‐height ratio is generally higher at a lower site productivity, but this trait is not strongly influenced by any environmental factor. This theoretical analysis brings into question established beliefs about the effects of latitude on crown shapes. By introducing geometry‐related growth constraints caused by shading from both the surrounding forest and the tree on itself, and costs for constructing and maintaining a three‐dimensional crown, our analysis suggests crown shapes may vary with latitude, mostly via effects on overall site productivity, and less because of the angle of the sun.

## 1 INTRODUCTION

Tree crown shapes vary considerably among and within terrestrial biomes. Characterizing the typical tree crown shapes of a biome is almost impossible, partly because tree crown shapes are inherently difficult to describe, and partly because the shapes vary depending on local growth conditions and tree age. Tropical and temperate forests are typically layered and dominated by tall top‐heavy trees, and the lower layers are more bush like. Boreal forests are dominated by conifers, but to describe pines, spruces, and larches as large cones, with most of the biomass close to the ground, would be overly simplistic. According to satellite data, most terrestrial forests are dominated by top‐heavy trees, that is, there is very little biomass close to the ground compared to higher up in the canopy (Lefsky, 2010).

Much effort has been invested in studies of the latitude and light influence on tree crown shape (Horn, 1971; Kuuluvainen, 1988, 1992; Mäkelä, 1985). The idea that crown shapes can be optimally shaped to assimilate light goes back to at least Jahnke & Lawrence (1965), who showed that for stand‐alone plants, high conical shapes were superior to low conical shapes when the light was not coming from directly above the plant. In a follow‐up study considering prolonged growth periods, Oker‐Blom and Kellomäki (1982) concluded that no optimal crown shape could be found at any latitude. Both long columnar crowns and flat disk crown were effective to the north and south. In a study of optimal crown architecture in nongrowing stands, Kuuluvainen (1992) found that to minimize self‐shading and mean‐field shading, it is optimal for a tree at high latitudes to have a narrow elongated crown, while closer to the equator a top‐heavy wide crown is optimal. We do not know whether these results apply also to the evolution of trees in size‐structured populations considering individuals of all sizes from juveniles to mature adults.

The first important steps into understanding crown shape evolution were taken by Iwasa, Cohen, and Leon (1985) using light competition models with a cylindrical crown shape and ignoring horizontal neighbor interactions. In this landmark study, Iwasa et al. (1985) derived evolutionarily stable tree heights and crown shapes by analyzing competition for light, in a frequency‐dependent selection resulting from the interaction of height strategies. As expected, they found that the evolved tree height in a monomorphic equilibrium increases with tree density and the amount of leaves per tree. Surprisingly, they also discovered that when the tree crown is thin enough, a polymorphic equilibrium becomes possible that encompasses trees with several different heights. Taking their investigation further, they were able to show that in the monomorphic equilibrium at which all trees have the same crown shape, all trees should have some foliage all the way to the ground. While this conclusion is in contrast to empirical observation, the authors offered several possible reasons for a cutoff height for foliage, where all leaves are above the cutoff height of the trunk. The study by Iwasa et al. (1985) is the only vegetation model found in a review by Falster and Westoby (2003) that was able to produce a polymorphic evolutionary equilibrium. Later, Yokozawa, Kubota, and Hara (1996) studied crown shape coexistence in an ecological time scale in a size‐structured model and found coexistence in some cases. A more recent example is Vermeulen (2014), who investigated the influence on light direction on the evolution of crown shapes, albeit in a forest without size structure. He showed that crown top‐heaviness (more precisely the crown ratio) was increasing, as the incident sun angle was decreasing.

Why are some tree architectures found in one climate but not in another? To answer this question, we have to consider abiotic factors such as light, temperature, and precipitation, but also interaction with other trees and organisms in the forest. Arguably, the most important interaction among tall plants is competition for light, as tall plants are the result of an evolutionary arms race for more light (Givnish, 1982; Iwasa et al., 1985). Without competition for light, it makes little sense to invest in costly trunks, but even without this competition, the optimal plant architecture will depend on the incident sun angle (Jahnke & Lawrence, 1965). Latitude has two effects on the light arriving at a leaf in the canopy resulting from: (a) The amount of vegetation that light traverses, and (b) The amount of atmosphere that light traverses. Sun light emitted at a lower incident angle, with the sun closer to the horizon, will both go through more atmosphere and more vegetation, compared to a higher sun angle. Both these shading effects depend on the movement of the sun during the day and throughout the year. The atmospheric effect also depends on if the air is clear or cloudy. Disentangling these two effects is important when trying to predict the latitudinal effect on tree crown shape.

Tree‐crown evolution with optimizing selection has previously been studied by Niklas (1999). The trees in his model are self‐similar, in contrast to the simple geometric objects and functions usually investigated in ecological tree models. A shape is self‐similar if it looks similar on a small and on a large scale, that is, it looks similar if you zoom in on a part of it. Niklas explored the fitness landscape using an evolutionary walk where the tree maximizes light interception, mechanical stability, reproductive capacity, and minimizes surface area (to minimize evaporation). These goals cannot in general be achieved simultaneously; Niklas found several plausible tree shapes when trying to balance these conflicting goals. When only light interception is maximized, the plants evolve toward a flat‐top tree, which minimizes self‐shading. This is, however, also the worst strategy for mechanical stability as the large horizontally extended branches have high bending gravitational force acting upon them, an effect that can be enhanced by snow cover and wind. Niklas work highlights the importance of considering trade‐offs in crown architecture. While his approach yields insights into factors shaping tree architecture, the definition of fitness in his model is statically prescribed rather than dynamically derived from an underlying ecological model. This is also true for more recent functional‐structural plant models which may have detailed light assimilation and growth, but usually do not consider the full lifecycle of plants including mortality and regeneration (Cournède, Mathieu, Houllier, Barthélémy, & De Reffye, 2007; Pearcy & Yang, 1996). The focus on optimizing selection neglects the endogenous environment that is created by the trees themselves and thereby prohibits the emergence and coexistence of different tree types through frequency‐dependent interactions.

While the studies by Iwasa et al. (1985) and Niklas (1999) were important achievements, neither account for the process of ontogenetic growth from seedlings to large trees. As tree‐crown shapes that are competitively superior in early phases of vegetative growth need not to be competitive during later phases, the ontogenetic growth process potentially has important evolutionary implications. A first inroad into understanding these implications has been provided by Yokozawa et al. (1996), who studied the coexistence of conic‐canopy plants (conifers) and spheroidal‐canopy plants (hardwoods). However, no study to date has considered the evolution of tree‐crown shape in a dynamic model of growing plants.

Here, we investigate how crown shape depends on latitude and site productivity, using a size‐structured model with light competition following Falster, Brännström, Dieckmann, and Westoby (2011). As latitude influences both the mean incident sun angle and the light‐response curve, we also investigate the sun angle and the light‐response curve separately. As the mean sun angle decreases with increasing latitude the forest becomes darker. The light‐response curve describes the instantaneous rate of CO_2_ assimilation depending on the incoming light intensity I, and the canopy openness (*E* = [0,1]). The amount of atmosphere that sun light traverses increases with increasing latitude decreases the light‐response curve. In order to find out if evolution is optimizing net primary production (NPP), we find the evolutionarily stable strategy (ESS) for the crown shape and compare it to the strategy maximizing NPP. Finally, we investigate correlations between crown shape, and NPP or leaf area index (LAI).

## 2 MATERIALS AND METHODS

**Figure 1:**
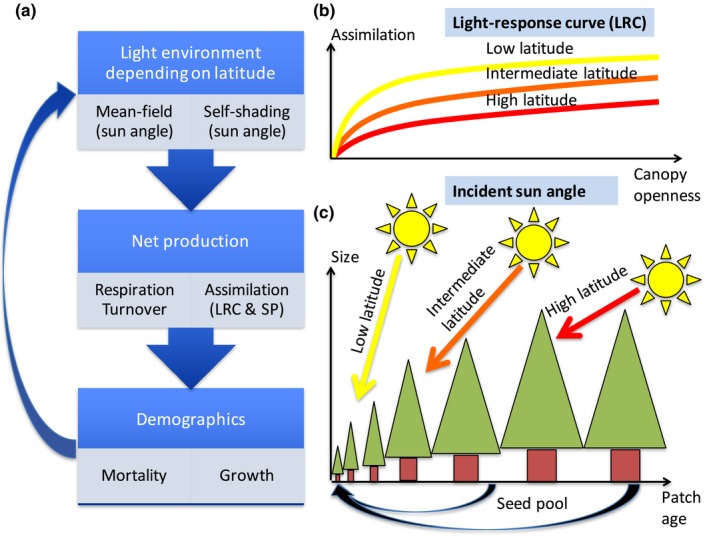
(a) Flow diagram showing the main parts of the stand model by Falster et al. (2011), and the influence of our additions: (b) the light‐response curve (LRC), (c) the incident sun angle, and the site productivity (SP). Latitude controls both the incident sun angle and the light‐response curve. Both the mean shading of the forest and the self‐shading produced of an individual tree are influenced by the latitude via the sun angle, and latitude is also influencing the light‐response curve describing the assimilation of CO_2_ depending on canopy openness. We use the weighted yearly mean of the sun angles at a specific latitude. The sun angles are weighted with the instantaneous light intensity during daytime at a horizontal plane. As the mean sun angle decreases with increasing latitude, the forest becomes darker. A higher latitude correlates with a generally lower incident sun angle, which means there is more atmosphere for the sun beams to traverse, and therefore, a higher latitude corresponds to a lower light‐response curve

The model is described in four subsections: (a) Environment, (b) Physiology, (c) Demographics, and (d) Evolution. We want to understand the crown shape evolution depending on site productivity and latitude. Both the light‐response curve and the incident sun angle depend on the latitude. For this purpose, we designed a size‐structured population model with growing trees, where the individual tree is described by a physiological model based on Falster et al. (2011). The self‐shaded tree crown shape is described by the top‐heaviness (*η*) and the width‐to‐height ratio (*ζ*). The mean‐field shading of light in the forest depends on the density of the individual trees (*n*), and there is only a vertical dimension of the forest. Disturbances remove all vegetation within a patch, and each patch contributes to the common seed pool depending on the age of the patch. The model is analyzed using adaptive dynamics (Dieckmann & Law, 1996; Geritz, Kisdi, Meszéna, & Metz, 1998; Metz, Geritz, Meszéna, Jacobs, & Van Heerwaarden, 1996; see also Brännström, Johansson, & von Festenberg, 2013 for an introduction), where a rare mutant strategy is competing against a common resident strategy. A schematic illustration of the model is given in Figure 1. Model equations and parameters are given in the Supporting Information Appendix S1.

### 2.1 Environment

Latitude influences a multitude of abiotic factors such as temperature and precipitation. We consider two factors related to latitude: the incident sun angle per se influencing self‐shading and mean‐field shading of the vegetation, and the effects of reduced ecosystem productivity at high latitudes caused by the atmosphere influencing the light‐response curve (Figure 1).

The light‐response curve describes the CO_2_ assimilation at different levels of canopy openness (*E*), and it depends on the amount of atmosphere the sun light traverses before arriving at the top of the canopy. A higher latitude correlates to a generally lower incident sun angle, which means there is more atmosphere for the sun beams to traverse, and therefore, a higher latitude corresponds to a lower light‐response curve (Figure 1). For each latitude, we construct a light‐response curve by simulating the sun movement during all hours and days of one year (Supporting Information Appendix S2).

The incident sun angle varies during the day, but also during the year. Here, for simplicity, we use the weighted yearly mean of the sun angles at a specific latitude. The sun angles are weighted with the instantaneous light intensity during daytime at a horizontal plane. For reference, the weighted mean sun angle is 19° at 90° Lat., and 55° at 0° Lat. The effect of the incident sun angle on self‐shading is depending on the shape of the tree, while the effect on the mean‐field shading is depending on the forest structure, that is, the density, shape, and height of all the individual trees (Supporting Information Appendix S3).

### 2.2 Physiology

**Figure 2:**
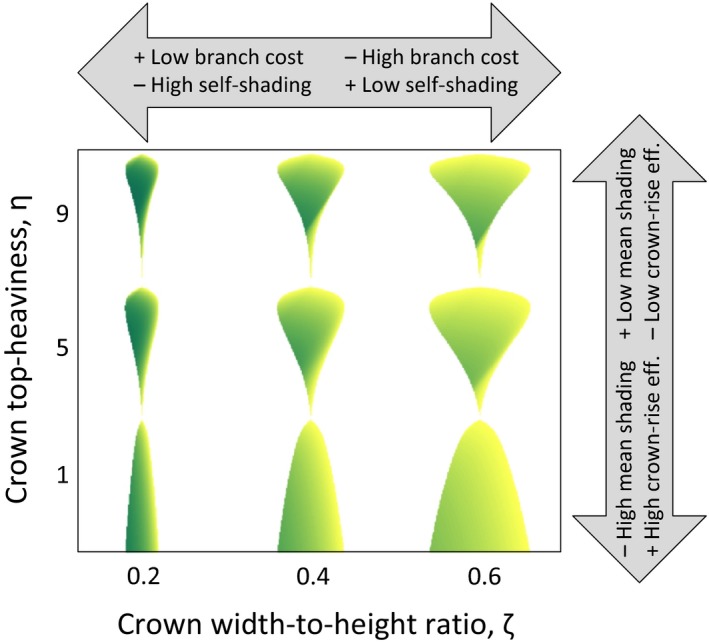
Light assimilation and crown shape trade‐offs. Characteristic tree‐crown shapes resulting from different values of the two evolving traits: crown top‐heaviness and crown width‐to‐height ratios. The trade‐offs for both traits are indicated in the block arrows. A wide crown has high branch costs, but the leaf density is lower and this decreases self‐shading and consequently increases light assimilation. A top‐heavy crown experiences low mean‐field shading from the surrounding forest, but crown‐rise efficiency is lower as the tree needs to discard branches in order to preserve the crown shape as the tree gains in height

We use the same framework as Falster et al. (2011) and extend it with three new features: self‐shading, an extended pipe model, and crown‐rise efficiency. The tree‐crown shape, described by the two traits *η* and *ζ*, influences all these features. The *η* trait describes the top‐heaviness of the plant. In a top‐heavy tree, most of the leaves are at the top half of the trunk. The *ζ* trait describes the crown width‐to‐height ratio (Figure 2).

Our model is individual based and size‐structured; in other words, we consider the full lifecycle of individual plants from seedling to mature trees interacting with individuals of all other sizes. Trees start growing at predetermined seed‐size (*s*) and grow until the height at maturation (*h*_*m*_) where growth is almost ceased. We only consider large trees. For simplicity, we assume that fine root, bark, heartwood, and sapwood masses are all depending on leaf mass. Mass allocation is dependent on the shape of the plants, described by the extended pipe model (Supporting Information Appendix S4). As the leaf mass increases, the mass allocation relation changes from almost only leaf mass to almost only heartwood mass and sapwood mass (Supporting Information Appendix S5). Growth is driven by net production which is the photosynthetic assimilation income minus respiration and turnover costs. For the assimilation, we consider both the self‐shading of the plant by itself and the mean‐field shading of the plant by the surrounding forest. Mean‐field shading is describing the vertical shading from the leaves that light passes through, more exactly the integrated leaf area above any given height in the canopy. Shade tolerance is not modeled specifically, but bottom‐heavy narrow tree requires less light to grow. Costs from respiration and turnover increase with plant size, as the mass fraction of supporting tissue is increasing (i.e., sapwood and heartwood). Also, the mortality is controlled by the net production ‐ if net production is too low the plant will die. The age distribution of patches is decreasing exponentially with age based on a gap dynamics model. The seed rain is calculated from the age‐dependent plant density distribution and the mass‐dependent fecundity. This seed rain is our fitness measure used in the evolutionary analysis.

Tree crown circumference is described by the Yokozawa function (Yokozawa & Hara, 1995)

$$q\left(y,h\right)=2\eta \left(1-y^{\eta }h^{-\eta }\right)y^{\eta -1}h^{-\eta },$$

where *y* is the height above ground, *h* is the height of the tree, and the top‐heaviness increases with *η*. This *η* is one of our two traits together with the crown width‐to‐height ratio *ζ*, where the crown width is the maximum tree width. As the tree crown shape is scaling isometrically, *ζ* is conserved during ontogenetic growth.

The self‐shading depends on the crown shape, the leaf area *ω* per crown volume *V* of the tree, and the sun angle. We assume that the crown shape is preserved up to isometric scaling during growth. The leaf area per volume μ(*η*,* ζ*) influences the light attenuation in the crown. We assume leaf area is proportional to leaf mass *m*_*l*_. We assume that the tree is illuminated from many directions at equally distributed azimuthal angles; therefore, it suffices to determine the light assimilation for each point in a two‐dimensional cross section of the tree crown containing the stem (Supporting Information Appendix S3).

We extend the pipe model by Shinozaki, Yoda, Hozumi, and Kira (1964a), Shinozaki, Yoda, Hozumi, and Kira (1964b) into a three‐dimensional setting that takes into account both vertical and horizontal pipes, in contrast to the original pipe model that only considers vertical pipes. The new pipe model has the effect of reducing the competitive fitness of trees with very wide crowns that would otherwise outcompete all other shapes as they have very low self‐shading. Wide crowns have a low self‐shading as the density of leaf mass is decreasing with increasing width. The pipe model theory, first proposed by Shinozaki et al. (1964a), Shinozaki et al. (1964b), assumed constant ratios between sapwood cross section and foliage mass. It applies to both stem and branches and assumes that a pipe from the ground supported every leaf unit. The integration over all the pipes is translated into mass of sapwood and heartwood. Heartwood is dead sapwood, which means that it is an effect of turnover, but in this model, we allocate directly to heartwood, as in the Falster et al. model (Supporting Information Appendix S4 and S5).

Crown‐rise efficiency describes how efficiently the crown rises during growth and in the simple form presented here it depends only on the crown shape and not the tree height, but there are other formulations depending on the height of the tree and the height of the canopy (Valentine, Amateis, Gove, & Mäkelä, 2013). We assume a fixed top‐heaviness and a constant width‐to‐height ratio of the trees during their lifetime. To maintain the shape, a top‐heavy crown has to drop leaves while growing, and a more top‐heavy shape will lose more biomass than a bottom‐heavy shape giving a lower crown‐rise efficiency. The crown‐rise parameter *k* describes the percent of productive biomass that is lost through crown rise, which includes both leaves and branches. Here, *k* = 1 means that no productive leaves or branches are lost due to crown rise; in other words, crown‐rise efficiency will be 100%. On the other hand, *k* = 0 means that only productive leaves and branches are lost, and this will result in a low crown‐rise efficiency depending on crown shape. Typically few leaves and branches are lost due to crown rise, as most leaves and branches are lost due to turnover, meaning that *k* should be close to 1 (Supporting Information Appendix S6).

We do not consider the different photosynthetic abilities of evergreen vs deciduous trees. However, these abilities are not clearly related to the crown shape as trees from both categories can vary widely in shape. Fertility is modeled as site productivity, the amount of photosynthesis per unit leaf area and light, and affects the assimilation (*A*). Note that the allocation to fine root mass is scaling with the individual plant leaf mass, and therefore indirectly depends on fertility.

### 2.3 Demographics

The model comprises two coupled components, with the first describing the age distribution of patches and the second describing the size distribution within patches. Patches only interact through random seed dispersal. Seeds enter a common seed pool and are then redistributed randomly among the patches. There is no horizontal structure within patches, meaning that trees are only interacting through a mean field. The probability *p*(*a*) of a patch remaining undisturbed for *a* years decreases monotonically with patch age, and the patch dynamics is described by the Von Foerster‐McKendrick's equation (Supporting Information Appendix S7). These disturbances could be caused by top‐down controls such as storms, fires, and grazing. We assume that the disturbances cause a total destruction of all vegetation in that patch. A patch is defined by its age. Within patches, the density of plants *n*(*η*,* ζ*,* m*_*l*_, *a*) with two‐trait strategy (*η*,* ζ*),leaf mass *m*_*l*_, and age *a* is governed by the equation

$$\frac{\partial }{\partial a}n\left(\eta ,\zeta ,m_{l},a\right)\;=\;-d\left(\eta ,\zeta ,m_{l},a\right)n\left(\eta ,\zeta ,m_{l},a\right)-\frac{\partial }{\partial m_{l}}\left[g\left(\eta ,\zeta ,m_{l},a\right)n\left(\eta ,\zeta ,m_{l},a\right)\right].$$

The boundary conditions define the density of plants at age zero, and the density of plants at leaf mass zero (*m*_*l*,0_)

$$n\left(\eta ,\zeta ,m_{l,0},a\right)\;=\;\frac{\pi_{1}\left(\eta ,\zeta ,m_{l,0},a\right)}{g(\eta ,\zeta ,m_{l,0},a)}y_{\eta ,\zeta },$$

$$y_{\eta ,\zeta }\;=\;\int_{0}^{\infty }p\left(a\right)\int_{0}^{\infty }\pi_{0}f\left(\eta ,\zeta ,m_{l},a\right)n\left(\eta ,\zeta ,m_{l},a\right)\;dm_{l}\;da,$$

and

$$n(\eta ,\zeta ,m_{l,0},0)\;=\;0.$$

The growth dynamic depends on the growth rate *g*(*η*,* ζ*,* m*_l_, *a*), mortality rate *d*(*η*,* ζ*,* m*_l_, *a*), seedling survival during dispersal π_0_, seedling survival during germination π_1_(*η*,* ζ*,* m*_l,0_, *a*), and rate of seed production *f*(*η*,* ζ*,* m*_l_, *a*). The resident strategy (*η*,* ζ*) has the seed rain *y*_*η*,*ζ*_ which is the rate of seed produced by the forest per square meter. The seed rain *y*_*η*,*ζ*_ is defined at the ecological equilibrium. We find the light environment (canopy openness, *E*) at the ecological equilibrium iteratively by first guessing a seed rain *y*_0_ and then solving the system using a semi‐implicit upwind finite‐difference scheme (Supporting Information Appendix S7). From this, we determine the seed rain *y*_1_ which we use as input for a second iteration, and so on until we get convergence. We found that convergence was fast for all the considered simulations. We define the invasion fitness for a mutant (*η*′, *ζ*′) by *F*, which is the logarithm of the basic reproduction ratio *R*_0_, where *R*_0_ is the mutant seed rain and resident seed rain ratio

$$F\;=\;\log\left(\frac{y_{\eta^{\prime },\zeta^{\prime }}}{y_{\eta ,\zeta }}\right).$$

Here, $y_{\eta^{\prime },\zeta^{\prime }}$ is the seed rain from the mutant strategy growing in the resident environment, with the same boundary conditions as the resident. *F* > 0 means that the mutant can invade and otherwise it cannot. In Supporting Information Appendix S8, we show that our *R*_0_ is equivalent to the commonly used basic reproductive ratio, as calculated by Falster et al. (2015, 2016).

### 2.4 Evolution

For the evolutionary analysis, we use techniques from adaptive dynamics (Dieckmann & Law, 1996; Geritz et al., 1998; Metz et al., 1996). Adaptive dynamics is based on the idea that the ecological and evolutionary timescales can be separated. It is further assumed that mutants are rare and do not influence the dynamics of the ecosystem. A central tool in adaptive dynamics is the canonical equation, which describes resident evolution in the direction of higher invasion fitness. The invasion fitness can be determined for any mutant strategy, and a “fitness landscape” is thereby created. This fitness landscape importantly depends on the resident strategy and is the landscape continuously shifts as the resident strategy evolves. Gradual evolution comes to a halt at so‐called singular strategies that are maxima or minima in the fitness landscape. The former corresponds to an evolutionarily stable strategy (ESS). In the latter case, two new strategies eventually form through evolutionary branching. Astute readers will note that singular strategies are further classified according to their convergence stability. In our study, all reported ESS's are convergence stable and we therefore omit to state this explicitly. We find the ESS at the intersection of the evolutionary nullclines for the two evolving traits top‐heaviness and width‐to‐height ratio. The nullclines are at the zero gradient of the fitness landscape in the direction of one trait (Supporting Information Appendix S9).

### 2.5 Analysis overview

**Figure 3:**
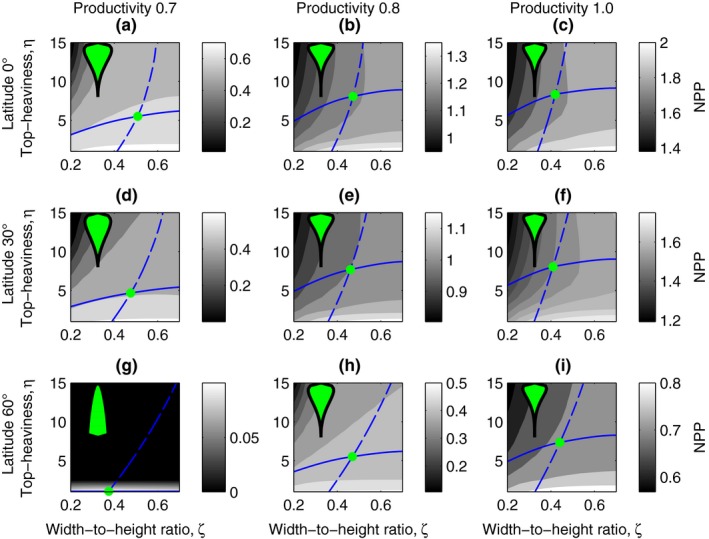
Crown shape ESS does not coincide with NPP maximum. Influence of latitude and site productivity on the evolutionarily stable (ESS) tree‐crown shape. Latitude influences both the shading effects from the incident sun angle (*δ*) and the light‐response curve (*c*_P1_ and *c*_P2_). The ESS (green dot) is at the crossing of the invasion fitness nullclines for crown width‐to‐height ratio (dashed blue line) and crown top‐heaviness (continuous blue line). The net primary production (NPP) is shown in the background, and the density of trees (*n*) correlates with the NPP. At high latitude and with low site productivity, we found the least top‐heavy tree crowns. The maximum NPP is not at the crown‐shaped ESS. Latitude is varied between 0° and 60°, and productivity between 70% and 100% (*p*_*s*_ = 0.7, 0.85, 1)

**Figure 4:**
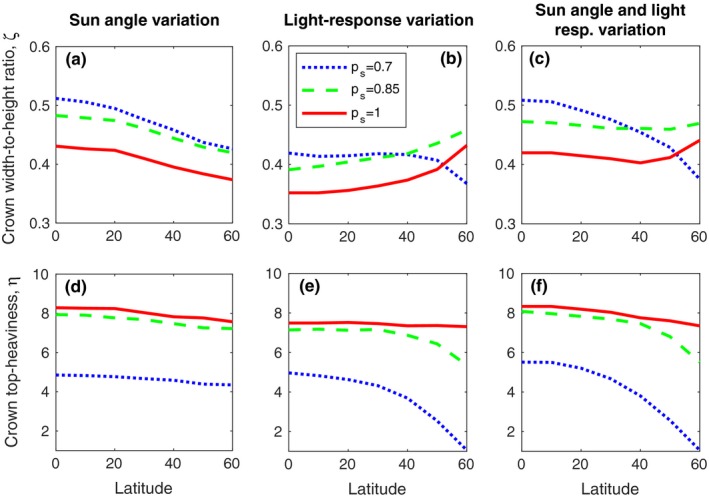
Only top‐heaviness is clearly decreasing with latitude. The tree crown shape ESS depending on latitude with shading effects from: (a,d) only incident sun angle (*δ*) changing with latitude, (b,e) only the light‐response curve (*c*_P__1_ and *c*_P__2_) changing with latitude, or (c,f) both factors changing with latitude. The factor not varied is fixed at its level on latitude 30°. Productivity is varied over *p*_*s*_ = 0.7, 0.85, 1

We investigate how the tree crown shape depends on two factors changing with latitude: (a) the sun angle influencing the self‐shading of an individual tree and the mean‐field shading of the forest, and (b) the amount of radiation arriving for a given level of shading described by the light‐response curve (the parameters *c*_P1_, and *c*_P2_). In Figure 3, both these two factors change with the latitude, while in Figure 4, we vary one factor at a time. We also vary an independent measure of site productivity (*p*_s_): the amount of photosynthesis per unit leaf area and light in Figures 3 and 4. To find the common evolutionarily stable strategy (ESS) of the two evolving traits, we first find the evolutionary nullclines, using a bisection method to find the zero invasion fitness gradient. Along the nullclines, the invasion fitness gradient is zero for one of the traits. When the nullclines cross the invasion fitness gradient is zero for both traits, that is, there is a common ESS for both traits. In our case, this strategy is also at the invasion fitness maximum, as there is only one ESS which is also convergence stable (Supporting Information Appendix S9).

## 3 RESULTS

### 3.1 Light assimilation and crown shape trade‐offs

First, consider the crown width‐to‐height ratio. As the leaf area *ω*(*m*_l_) is proportional to the leaf mass *m*_*l*_, which is held constant in Figure 2, the leaves will be more densely packed when the tree‐crown volume is small. This implies that self‐shading is reduced by increasing the crown width‐to‐height ratio, as the crown volume increases. On the other hand, sapwood and heartwood mass, defined by the extended pipe model, will increase for larger crown width‐to‐height ratio, and this will decrease growth speed. Thus, the crown width‐to‐height ratio induces a trade‐off between light assimilation and costs for branches. Next, consider the crown top‐heaviness. As more of the leaf area is moved toward the top of the crown, the leaves will be more illuminated whenever the tree grows in a forest environment. With top‐heavy crowns, tall trees may largely avoid the shade in the deep canopy. This comes at the cost of higher branch mass, however, as the distance of the average pipe connecting leaves to the ground increases. There is also a further, less obvious, cost of top‐heavy tree crowns. As the crown rises throughout ontogenetic development, the lower branches will frequently need to be discarded in order to maintain the top‐heavy crown shape. Hence, also crown top‐heaviness induces a trade‐off between light assimilation and costs for branches, though for the latter the dominating factor is the lower crown‐rise efficiency.

The above argument suggests that crown width‐to‐height ratio is under optimizing selection, whereas crown top‐heaviness is under frequency‐dependent selection. While this may be largely true, we caution that the negative consequences of self‐shading and the acceptable costs for branch mass do depend on the surrounding environment. In particular, self‐shading at lower branches may not matter much if these are anyway located in a deep canopy shade.

We generally find strong selection for top‐heavy crowns, which is eventually counteracted by a decrease in crown‐rise efficiency. The increasing sapwood and heartwood mass generated by the increasing pipe lengths cannot themselves balance the selection for more top‐heavy crowns. The crown width‐to‐height ratio trait is less influenced by the environment.

### 3.2 Crown shape ESS does not coincide with NPP maximum

Recall that latitude in our model determines the incident sun angle and the light assimilation curve. Figure 3 shows selection for crown top‐heaviness and crown width‐to‐height ratio changes under nine combinations of latitude and site productivity. The solid and dashed line are evolutionary nullclines where selection for crown top‐heaviness and crown width‐to‐height ratio, respectively, ceases. The evolutionarily stable (ESS) crown shape is located at the intersection of the nullclines and is indicated with a green marker. We find that the evolved crown shape reaches a final top‐heaviness at low latitudes (close to the equator) and under high productivity (Figure 3b,c,e,f). It is not clearly visible how the width‐to‐height ratio is influenced by productivity and latitude, but see Figure 4. In the background of Figure 3, we show for comparison the net primary production (NPP). The ESS crown shape is generally far away from the maximum NPP, which implies that evolution is not maximizing NPP. Note that top‐heavy crowns evolve at low site productivity levels and high latitudes ‐ resulting in a very low NPP ‐ showing the strong effect of light competition. To verify that the evolved crown shapes are evolutionarily stable, we show in Supporting Information Appendix S9 that the invasion fitness for rare mutant strategies is always negative when growing among trees with the ESS crown shapes in Figure 3.

### 3.3 Only top‐heaviness is clearly decreasing with latitude

To determine whether the trend toward more top‐heavy tree crowns at lower latitudes is a consequence of the increased incident sun angle or the increased light income, we next investigate the evolution of crown shape while varying these two factors separately, at different levels of site productivity. In Figure 4a,b, we plot the evolutionarily stable (ESS) crown shape as a function of latitude when the light‐response curve and the average sun angle are, respectively, held constant to their corresponding values at latitude 30°. These outcomes, shown for three levels of site productivity, should be compared with Figure 4c in which both factors are allowed to change with latitude as described in Methods: Environment.

When only sun angle changes with latitude, and the light‐response curve is fixed at latitude 30°, the ESS crown width‐to‐height ratio (Figure 4a) and ESS crown top‐heaviness (Figure 4d), declines with latitude, at all site productivities. Crown width‐to‐height ratio decreases with increasing latitude when site productivity is low (*p*_s_ = 0.7), but instead at higher productivities (*p*_s_ = 0.85, 1), crown width‐to‐height ratio increases with latitude (Figure 4b), when only the light‐response curve changes with latitude, and incident sun angle is fixed at latitude 30°. By contrast, crown top‐heaviness decreases with latitude at all levels of site productivity (Figure 4e). When both sun angle and light response are allowed to change with latitude, the crown width‐to‐height ratio decreases with latitude at low site productivity (*p*_*s*_ = 0.7), but is almost independent of latitude at higher site productivity (*p*_s_ = 0.85, 1). By contrast, crown top‐heaviness decreases with latitude at all levels of site productivity, and especially at low site productivity (Figure 4f). In all scenarios, we find that the crown top‐heaviness decreases with site productivity, and that the crown width‐to‐height ratio has a more complex response to site productivity.

## 4 DISCUSSION

We evolved the tree‐crown shape in a stand with ontogenetic growth, with both self‐shading and mean‐field shading. We found that the evolved crown shape was always far away from the maximum NPP (Figure 3), as expected, as light competition forces trees to invest in large costly trunks, which are far from optimal. We investigated how the crown‐shaped ESS changed under variations in latitude and site productivity (Figure 3). Generally, the trends of evolved crown top‐heaviness were more straightforward to interpret than those for crown width‐to‐height ratio. A lower site productivity always resulted in a lower crown top‐heaviness at ESS, which can be explained by a lower density of trees decreasing the light competition (Figure 4d–f). Also, the crown width‐to‐height ratio was strongly affected by site productivity, and generally, we found a higher crown width‐to‐height ratio at a lower site productivity (Figure 4a–c). In total, this means that we expect wider and less top‐heavy trees to evolve when site productivity is low. Latitude had a weak effect on the crown shape, and only top‐heaviness was clearly decreasing with latitude at low site productivity (Figure 4e).

The most surprising findings of our study are the strong evolutionary tendency toward top‐heavy tree crowns, and the monomorphic ESS. Crown top‐heaviness increases the tree's ability to compete for light. This causes crown top‐heaviness to evolve in almost all light environments, except the very unproductive (Figure 3g), despite the associated costs from low crown‐rise efficiency and large sapwood and heartwood mass. These large supporting masses imply a large respiration and turnover, which lower the net growth rate. We found that this decrease in net growth rate related to supporting masses could not alone balance the race toward higher and higher top‐heaviness, and that an evolutionary balance was reached only when crown‐rise efficiency was also included. For top‐heavy crown shapes, the loss of biomass during crown rise is large as the branches cannot be moved upwards during growth; instead, lower branches are discarded and higher branches are grown.

On the other hand, the benefit of increasing the crown width‐to‐height ratio is that it reduces self‐shading, but this only has an effect if mean‐field shading is weak. A wider tree has more sparse leaves, which facilitates light penetration through the canopy. This explains why we generally found a higher crown width‐to‐height ratio when site productivity was low (Figure 4a–c), except at high latitudes. We found only monomorphic populations at the ESS, probably as all trees have the same height at maturation. It is known from theoretical models that trees at different heights can coexist in evolutionary timescales (Falster, Brännström, Westoby, & Dieckmann, 2017; Iwasa et al., 1985). Light competition is one of very few imaginable reasons for plants to grow high. Fire and grazing might also drive plants to evolve greater heights, but not more than say 10 m. Trees in most forests are much higher, and therefore, we can assume that tree crown shape is mostly affected by light limitation. Wind and snow are other important factors influencing tree crown shape.

Our results are consistent with Kuuluvainen's (1992) who found that a flat horizontally extended crown was optimal at low latitudes with high incident sun angle, while a narrow elongated crown is optimal at high latitudes with low incident sun angle, for uniform and nongrowing tree stands (c.f., Figure 4a,d). In real forests, stands are not usually uniform and static, but Kuuluvainen's results are still interesting and relevant. Recently, Vermeulen (2014) investigated an evolutionary model of nongrowing trees competing for light and found, in contrast to Kuuluvainen, that low sun angles resulted in the evolution of shallow crowns (i.e., top‐heavy crowns) and vice versa. Our model does not support the results by Vermeulen, as decreasing the sun angle always gives narrower, less top‐heavy trees. This suggests the importance of including detailed demographic and physiological processes. Further work will hopefully help to identify the factors and assumptions that cause these apparent contradictory results.

In their study on the effects of crown shape, Yokozawa et al. (1996) reported coexistence over ecological time scale of trees with conical and spherical crowns. Surprisingly, the Yokozawa et al. study also found that conic‐canopy plants could not persist if spheroid‐canopy plants were established first, contrary to field observations that spruce is a strong competitor in an established birch forest. As Yokozawa et al. described crown shape by the same one‐parameter function as we used to describe top‐heaviness, one might expect to see the same outcome also in our model. We tested for coexistence for two tree species with the same crown shapes assumed by Yokozawa et al., but we could not support this conclusion (results not shown). One possible explanation is that Yokozawa et al. studied coexistence only over a limited time span (500 days) and therefore did not observe the extinction that occurred on longer time frames. Our model also, in principle, allows for evolutionary diversification and the emergence of two or more coexisting strategies, but we did not observe this outcome under any scenario considered. While this provides some evidence to the belief that crown shape is not a central factor for species richness, we caution that diversification in crown shape may well occur in coevolution with other traits or under conditions not considered in our study.

Gaps are crucial for the regeneration of forests, as usually saplings can grow only here. It is difficult to test how important gaps are for upholding diversity, as all forests have gaps (Hubbell et al., 1999). More light going through gaps in the forest can be approximated by a lower light extinction coefficient, but this is a poor approximation as it does not account for the heterogeneous light environment created by gaps. The mean‐field model therefore cannot describe the forest light environment accurately without modifications, and so, for example, the Perfect Plasticity Approximation has been developed to overcome this problem (Strigul, Pristinski, Purves, Dushoff, & Pacala, 2008). In our model, we use a disturbance model to approximate gap dynamics, where the total reproductive output of the model is described by a common seed pool. Here, the seeds are coming from forest of all ages, and the contribution from each patch is decreasing with age.

In our model, we do not consider plastic responses which may be important for the crown to grow efficiently. However, some aspects of the tree crown shape appear to be invariant. For example, it is a common assumption that the highest trees in the top‐canopy and the lowest understory trees have different shapes due to different lighting conditions, but the crown ratio (crown width/crown height) has been shown to be independent of the tree size in many studies on tropical forests (Iida et al., 2011; Sterck & Bongers, 1998; Sterck, Bongers, & Newbery, 2001).

Understanding spatiotemporal patterns of tree crown shapes on a large scale is hard, because of confounding factors such as glaciation and slow recolonization, and because of the long generation times of trees. These types of slow processes mean that we typically do not observe terrestrial ecosystems at equilibrium. Nevertheless investigating the mechanisms that shape tree crowns in vertical light environments at equilibrium is an important first inroad toward understanding the evolution of tree crown shape in more complex and realistic settings.

## 5 CONCLUSION

Our results show that top‐heavy crowns will evolve in all possible light conditions and site productivities, except at very low site productivity. Site productivity seems especially important for the evolution of crown top‐heaviness. Latitude and incident sun angle have only weak effects on the crown shape, except at low site productivity where top‐heaviness is clearly influenced by latitude. This latitude influence is mainly caused by the lower light‐response curve at higher latitudes related to atmospheric shading, and not by the lower incident sun angle related to vegetation shading. Such insights are valuable for understanding how trees under selection pressure will respond to changes in the environment and will help us build better future vegetation models. An interesting extension would be to model plastic crown shapes, which might promote the coexistence of tree crown shapes. Another interesting extension would be to study to coevolution of tree height and crown shape, as polymorphic populations were found in previous models considering tree height evolution.

## CONFLICT OF INTEREST

None declared.

## AUTHOR CONTRIBUTIONS

M.L., U.D.., and Å.B. planned and designed the research. M.L. carried out the research. D.F. and L.Z. contributed modeling and ecological expertise. M.L., D.F., L.Z., U.D, and Å.B. discussed the results and wrote the manuscript.
