# Supplementary material for: Latitudinal effects on crown shape evolution
Source: Ecol Evol. 2018 Jul 22;8(16):8149–58. doi: 10.1002/ece3.4275 (PMC6144995; doi:10.1002/ece3.4275)
Supplement: Supplementary file 1 [file ECE3-8-8149-s001.pdf]

Supporting information

In Supporting information S1 we present the full details of our model with equations and parameters. In S2, we describe how self-shading is determined. In S3, we describe our three-dimensional extension of the pipe model. In S4, we present how mass allocation depends on plant height. In S5, we describe the numerical method used to resolve patch dynamics, which is based on an upwind scheme. In S6, we briefly describe crown-rise efficiency. In S7, we determine the light-response curve from data on solar movements. In S8 we prove that the quotient of the mutant seed rain and the resident seed rain is equivalent to the basic reproduction number, and therefore can be used as a measure of invasion fitness. Finally, in S9 we show graphically that the resident strategy is always evolutionarily stable.

1 Supporting information S1. Traits, equations and parameters.

(Tables in separate file "Supporting info tables" should go here.)

2 Supporting information S2. Light-response curve

The light intensity at ground depends on the sun elevation angle. This angle affects both the amount of atmosphere that light has to pass before hitting the ground, and the angle at which it hits the ground. We find the sun elevation at day d, time of the day t, and latitude ψ from three sun movement equations (in degrees), based on basic equations which can be found for example in the WINPHOT 5.0 documentation by Hans ter Steege, assuming that the altitude is at sea level, and neglecting the effect of longitude:

(1) Solar declination, the angle between the earth-sun line and the equatorial plane

$$\delta = 0.39785 \cdot \sin\left(4.869 + 0.0172 \cdot d + 0.03345 \cdot \sin(6.224 + 0.0172 \cdot d)\right).$$

(2) Hour sun angle, the angular distance that earth has rotated in a day

$$\eta = 15(t - 12).$$

(3) The sun elevation angle α, the angle between the horizontal plane and a line connecting to the sun, where ψ is the latitude

$$\sin\alpha = \sin\psi \cdot \sin\delta + \cos\psi \cdot \cos\delta \cdot \cos\eta.$$

The atmospheric effect depends on the transmissivity τ of the path that light travels in the atmosphere, and this is 0.7 – 0.8 on a clear day, but around 0.5 - 0.6 on a cloudy day. Here we set transmissivity to a default value of 0.8, corresponding to clear sky. The optical air mass $M(\alpha)$ is an estimate of the number of atmospheres that light traverses ($M(90) = 1$), and it depends on the sun elevation angle α as

$$M(\alpha) = \frac{1}{\cos(90 - \alpha)}.$$

The light intensity (or photon flux) for a leaf lying horizontally on the ground is

$$I = I_0 \tau^{M(\alpha)} \sin(\alpha) p w,$$

where $I_0 = 1360$ W is the incoming sun radiation energy outside the atmosphere at Earth, p is the fraction of light that is photosynthetically active, and w is converting from W $m^{-2}s^{-1}$ to μmol $m^{-2}s^{-1}$.

We simulate the sun movement during the year and from this approximate the parameters of the light response curve. The light response curve is defined as in Appendix A6 in Falster *et al.* (2011), where the instantaneous rate of CO_2 can be approximated by the rectangular hyperbola

$$A_{\text{inst}}(I, E, A_0\nu) = 0.5\Theta^{-1}\left(\Phi IE + A_0\nu - \sqrt{(\Phi IE + A_0\nu)^2 - 4\Theta\Phi IEA_0\nu}\right)$$

where E is the canopy openness, Φ is the quantum yield of assimilation (0.04 mol CO_2 mol photon^{-1} s^{-1}, Θ is the curvature factor ($= 0.5$), and $A_0\nu = A_{max} = 10.6029$ μmol s^{-1} m^{-2} is the ratio of light saturated CO_2 assimilation to leaf area with $A_0 = 5.67 \times 10^3$ μmol s^{-1} kg^{-1}, and $\nu = 1.87 \times 10^{-4}$ kg m^{-2}. The photon flux I was simulated using the three sun movement equations above. Integrating over the yearly photon flux, the relation between gross assimilation and canopy can be approximated by the light response curve

$$A_{\text{lf}}(E, A_0\nu) = c_{\text{P1}} \frac{E}{E + c_{\text{P2}}}$$

where the values for c_{P1} and c_{P2} depend on the latitude, and $E \in [0, 1]$. In practice we integrate over all day for a discrete set of days for a discrete set of canopy openness, where the integration kernel is $c_{\text{ext}}IE$. We multiply with light extinction c_{ext} to take into account that light extinction is equivalent to light absorption, all light that is extinct is absorbed.

In the model calculations we used these parameter values estimated from the solar movement simulations outlined above. Lat 0°: c_{P1}=169.8, c_{P2}= 0.2400; Lat 30°: c_{P1}=168.3, c_{P2}=0.2542; Lat 60°: c_{P1}=154.9, c_{P2}=0.3266.

3 Supporting information S3. Crown openness and self shading

We calculate the solar elevation angle at given latitude based on the mean sun angle above the horizon during a year, weighted with light intensity. We assume that the

tree is illuminated from many directions at equally distributed azimuthal angles. As a consequence of the latter assumption, it suffices to determine the light assimilation for each point in a two-dimensional cross section of the tree crown containing the stem. The total light assimilation is then found by integration with each point weighted by the circumference formed by rotation around the stem. This reduction to a two-dimensional problem simplifies the calculation since the two-dimensional cross-sectional area can be triangulated with the Matlab mesh function 'initmesh'. To optimally distribute the triangles we first distribute the points used in the 'initmesh' function equidistantly along the tree shape. By precalculating the crown openness we can more efficiently find a solution of the population PDE. Next we show the prodecure of this precalculation.

For a given direction of incoming light, the crown openness (i.e. self shading) at a point y in the tree crown depends on the distance $d_1(\eta, \zeta, y, v)$ that a light ray with direction v travels through the crown before reaching the point y, the leaf area volume density $\mu(\eta, \zeta)$, and the light-absorption coefficient c_{ext}, through the Beer-Lambert law,

$$E_{\text{s}}(\eta, \zeta, y, m_{\text{l}}, v) = \exp(-c_{\text{ext}}\mu(\eta, \zeta)d_1(\eta, \zeta, y, v)).$$

We discretize the incoming light across ten equidistant directions and take the average of $d_1(\eta, \zeta, y, v)$ across each direction v to arrive at $d(\eta, \zeta, y)$. We use this $d(\eta, w, y)$ to approximate the crown openness for all masses m_{l},

$$E_{\text{s}}(\eta, \zeta, y, m_{\text{l}}) = \exp(-c_{\text{ext}}\mu(\eta, \zeta)d(\eta, \zeta, y)).$$

4 Supporting information S4. Extended pipe model

When the plant is growing, the proportions of the mass that is leaf, root, and stem change (Falster *et al.*, 2011). We derive the mass of sapwood from a three-dimensional pipe model, assuming that every leaf of the tree is supported by water and nutrients from the ground. The crown shape, or radius at height z, for a tree of height h,

$$S(\eta, \zeta, z, h) = w(\eta, \zeta)h^{3/2}\sqrt{\frac{q(\eta, z, h)}{\pi}},$$

is expressed using the leaf area density $q(\eta, z, h)$, where z is the height above ground and h is the height of the tree. Here w is the width scaling of the crown. When $S(\eta, \zeta, z, h)$ is rotated around the z axis we get the volume of the tree crown $V_{\text{c}}(\eta, \zeta, m_{\text{l}}) = h(m_{\text{l}})^3 w(\eta, \zeta)^2$, because the leaf area density $q(\eta, z, h)$ integrated over 0 to h is equal to 1. The tree crown volume scales with height as h^3, and scales with the width-to-height trait as w^2. Each volume element $\Delta V(x, y, z)$ in the crown with leaf mass $\Delta m_{\text{l}} = \mu(\eta, \zeta)\phi\Delta V$ is assumed to be supported by a pipe from the ground of length $l(x, y, z)$, and cross-sectional area ΔA [m^2]. Here $\mu(\eta, \zeta)\phi$ [kg m^{-3}] is the leaf mass density per volume, and ϕ is the leaf mass per area.

We assume that water flow rate per unit sapwood area is r [m h^{-1}] and that the water consumption per unit leaf mass is b [m^3 h^{-1} kg^{-1}]. Then

$$r\Delta A = b\mu(\eta, \zeta)\phi\Delta V.$$

For the whole tree trunk, we have the sapwood cross-sectional area A which is related to the tree crown volume as

$$rA = b\mu(\eta,\zeta)\phi V = bm_{\mathrm{l}},$$

$$A = \frac{bm_{\mathrm{l}}}{r}. \tag{1}$$

Sapwood area is assumed to be proportional to leaf area ω as

$$\theta = \frac{\omega}{A} = \frac{m_{\mathrm{l}}}{\phi A}. \tag{2}$$

We find from (1) and (2) that

$$\frac{b}{r} = \frac{1}{\theta\phi}.$$

This quotient will be used later. The volume of a pipe connecting to leaf area at a point in the tree crown with Cartesian coordinates (x, y, z) is

$$\Delta V_{\mathrm{p}} = l(x, y, z)\Delta A = l(x, y, z)k\Delta V,$$

with $k = b\mu(\eta,\zeta)\phi/r = \mu(\eta,\zeta)/\theta$ [m^{-1}]. The total volume of the pipes in the crown is thus

$$V_{\mathrm{p}} = \int_K dV_{\mathrm{p}} = k\int_K l(x, y, z)\ \mathrm{d}V,$$

where K is the domain of the tree crown. Now rewrite the integral using polar coordinates

$$V_{\mathrm{p}} = k\int_0^h \int_0^{S(z,h)} \int_0^{2\pi} l(z, r)r\ \mathrm{d}r\ \mathrm{d}\theta\ \mathrm{d}z,$$

where $S(z, h)$ is the radius of the tree crown at height z for a tree of height h. The length of the pipe is independent of the angle θ because the tree is assumed symmetric around the vertical z axis. We need to make an assumption about the architecture of the tree in order to determine the pipe. As the shortest path $l(z, r) = \sqrt{z^2 + r^2}$ will not result in a trunk, but rather a structure akin to a cone standing on its apex, we make the more realistic assumption that the pipe first connects horizontally from the leaf to the stem and then vertically along the stem to the ground, i.e., we assume that $l(z, r) = r + z$. The total pipe volume is then

$$V_{\mathrm{p}} = k\int_0^h \int_0^{S(z,h)} \int_0^{2\pi} (r + z)r\ \mathrm{d}r\ \mathrm{d}\theta\ \mathrm{d}z,$$

which we simplify to

$$V_{\mathrm{p}} = 2\pi k\left(\int_0^h \frac{S(z,h)^3}{3}\ \mathrm{d}z + \int_0^h z\frac{S(z,h)^2}{2}\ \mathrm{d}z\right) = I_1 + I_2.$$

We evaluate I_1 numerically as,

$$I_1 = 2\pi k\int_0^h \frac{S(z,h)^3}{3}dz = \frac{2^{5/2}\mu(\eta,\zeta)w^2h^4}{3\sqrt{\pi}\theta}\int_0^1 \left[\eta(1-x)^\eta x^{\eta-1}\right]^{3/2}\ \mathrm{d}x,$$

while I_2 has an analytic expression,

$$I_2 = 2\pi k \int_0^h z \frac{S(z,h)^2}{2} dz = \frac{kw^2h^4}{2}\left(1 - \frac{2}{1+\eta} + \frac{2}{1+2\eta}\right) = \eta_{\mathrm{c}}\mu(\eta,\zeta)\theta^{-1}w^2h^4.$$

Here we have defined

$$\eta_{\mathrm{c}} = 1 - \frac{2}{1+\eta} + \frac{2}{1+2\eta}.$$

We are now ready to formulate the mass of sapwood

$$m_{\mathrm{s}} = \rho\eta_{\mathrm{s}}\mu(\eta,\zeta)\theta^{-1}w^2h^4,$$

where we have defined

$$\eta_{\mathrm{s}} = \eta_{\mathrm{c}} + \frac{2^{5/2}w}{3\sqrt{\pi}} \int_0^1 \left[\eta(1-x)^\eta x^{\eta-1}\right]^{3/2} \,\mathrm{d}x.$$

Note that both η_{c} and η_{s} are dimensionless. The mass of heartwood is also dependent on η_{s}

$$m_{\mathrm{h}} = \rho\eta_{\mathrm{s}}\alpha_2\left(\frac{\omega(m_{\mathrm{l}})}{\omega_0}\right)^{\beta_2},$$

as well as the mass of bark

$$m_{\mathrm{b}} = bm_{\mathrm{s}}.$$

5 Supporting information S5. Mass allocation

When a tree has positive net production, the surplus biomass that remains after investments in reproduction is allocated to either of five compartments: leaf, fine roots, bark, sapwood, and heartwood. Figure S1 shows how the relative amounts of biomass in the four living compartments change with tree height. A sapling consists mostly of leaves and sapwood. As it grows into a large tree, sapwood quickly becomes the by far largest living compartment by weight. The allocation to the compartments is determined by allometric relationships in Table S1: Individual state.

6 Supporting information S6. Crown-rise efficiency

Here we derive the crown-rise efficiency, which depends only on the shape of the crown. The crown-rise efficiency is related to self-pruning, which depends on the shading of the forest in Mäkelä (1997). In our model the forest shading and the crown-rise efficiency influences the growth of the leaf mass of an individual tree. The crown-rise efficiency of a tree growing from leaf mass m_1 to leaf mass m_2 is defined as

$$C(m_1,m_2) = \frac{m_2 - m_1}{m_2 - m_1 + (1-k)f(m_1,m_2)},$$

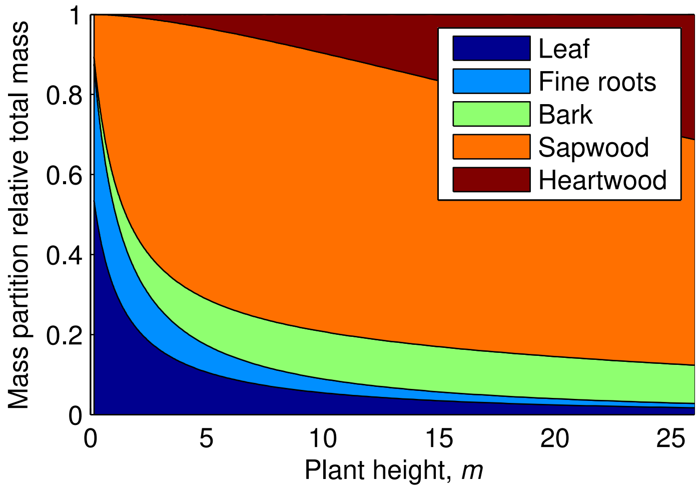

Figure S1. Mass partitioning for a top-heavy tree shape ($\eta = 12$) as a function of plant height. Masses for leaf, bark, fine roots, sapwood, and heartwood are shown relative to total living biomass.

where $f(m_1, m_2)$ is the discarded leaf and branch mass during the growth phase and $(1-k)$ is the fraction of this lost biomass that is productive tissue. If $f(m_1, m_2) = 0$ the crown-rise efficiency is 1, or 100%, but in general some mass is lost during growth. In the following derivation we assume that (i) tree growth is isometric (shape-preserving), (ii) all biomass lost is in the form of branches, and (iii) $f(m_1, m_2)$ excludes regular turnover of branches. Furthermore we assume the Yokozawa crown shape function (Yokozawa & Hara, 1995)

$$q(z, h) = 2\eta(1 - z^{\eta}h^{-\eta})z^{\eta-1}h^{-\eta}$$

describing the vertical density of leaf depending on the height above ground z. The height function

$$h(m) = \alpha m^{\beta}$$

relates tree mass m to tree height, where α and β are scaling constants. If we ignore the horizontal distribution of leaf and only look at the vertical distribution leaf mass will only be lost under the height z^* because of the simple geometry of the Yokozawa function, and therefore we can write

$$f(m_1, m_2) = \int_0^{z^*} g(m_2, z) - \int_0^{z^*} g(m_1, z)\mathrm{d}z = \int_0^{z^*} g(m_2, z) - g(m_1, z)\mathrm{d}z$$

where $g(m, z) = mq(z, h(m))$. Divide by Δm and take the limit $m_2 \to m_1$ from above with $m_1 = m$ and $m_2 = m + \Delta m$

$$\lim_{\Delta m \to 0} \frac{f(m, m+\Delta m)}{\Delta m} = -\lim_{\Delta m \to 0} \frac{\int_0^{z^*} g(m+\Delta m, z) - g(m, z)}{\Delta m}\mathrm{d}z = -\int_0^{z^*} \frac{\mathrm{d}g(m, z)}{\mathrm{d}m}\mathrm{d}z$$

The minus sign is there because mass can only be lost not gained. Using the Yokozawa function we get

$$g(m,z) = mq(z,h(m)) = 2m\eta(1 - z^{\eta}h^{-\eta})z^{\eta-1}h^{-\eta}.$$

We take the derivative of $g(m,z)$ and when $\frac{\mathrm{d}g(m,z)}{\mathrm{d}m} = 0$ we find the end point of the integral interval

$$z^*(m) = h(m)\left(\frac{1-\eta\beta}{1-2\eta\beta}\right)^{1/\eta}.$$

Next we integrate

$$\int_0^{z^*} \frac{\mathrm{d}g(m,z)}{\mathrm{d}m}\mathrm{d}z = 2\left[(1-\eta\beta)\left(\frac{z^*}{h(m)}\right)^{\eta} + \frac{1}{2}(2\eta\beta - 1)\left(\frac{z^*}{h(m)}\right)^{2\eta}\right].$$

This expression is independent of mass since

$$\frac{z^*(m)}{h(m)} = \left(\frac{1-\eta\beta}{1-2\eta\beta}\right)^{1/\eta}.$$

This also means that the crown-rise efficiency is indepent of mass - at least in this case. We can see this from $\mathrm{d}m = m_2 - m_1$ and

$$C(m_1,m_2) = \frac{m_2 - m_1}{m_2 - m_1 + f(m_1,m_2)} = \frac{\mathrm{d}m}{\mathrm{d}m + f(m, m+\mathrm{d}m)}.$$

By dividing by $\mathrm{d}m$ in the nominator and denominator and letting $\mathrm{d}m \to 0$ we get

$$C = \frac{1}{1 - \int_0^{z^*} \frac{\mathrm{d}g(m,z)}{\mathrm{d}m}\mathrm{d}z}.$$

Multiplying the second term of the denominator with the loss factor $(1-k)$, which represents the fraction of lost biomass that is productive biomass, we get the final expression

$$C(\eta) = \frac{1}{1 + 2(1-k)\left[(1-\eta\beta)\frac{1-\eta\beta_1}{1-2\eta\beta_1} + \frac{1}{2}(2\eta\beta - 1)\left(\frac{1-\eta\beta_1}{1-2\eta\beta_1}\right)^2\right]}.$$

7 Supporting information S7. Numerical method

We use a semi-implicit upwind finite-difference scheme for solving the size-structured population model within a patch. First introduce the following notations: density of individuals $n_j^i = n(t_i, m_j)$, growth $g_j^i = g(t_i, m_j)$, birth rate $f_j^i = f(t_i, m_j)$, and mortality $d_j^i = d(t_i, m_j)$. Then the McKendrick-von-Foerster equation can be discretized as

$$\frac{n_j^{i+1} - n_j^i}{\Delta t} + \frac{g_j^i n_j^{i+1} - g_{j-1}^i n_{j-1}^{i+1}}{\Delta m_j} = d_j^i n_j^{i+1},$$

where i is the time index and j is the mass index ($0 \leq j \leq J$), and Δt is the time increment and $\Delta m_j = m_j - m_{j-1}$. The boundary condition can be approximated by

$$g_0^i n_0^{i+1} = R(\mathbf{F}^i, \mathbf{n}^i),$$

where $\mathbf{F} = (f_0^i, \cdots, f_J^i)$, $\mathbf{n} = (n_0^i, \cdots, n_J^i)$, and $R(\mathbf{F}^i, \mathbf{n}^i)$ is the flux of new recruits at previous time step, which is approximated using the composite trapezoidal rule. Here the new recruits correspond to the seed rain.

We rearrange the discretized McKendrick-von-Foerster equation to

$$n_{j-1}^{i+1}\left(-\frac{\Delta t}{\Delta m_j} g_{j-1}^i\right) + n_j^{i+1}\left(1 + \frac{\Delta t}{\Delta m_j} g_j^i + \Delta t d_j^i\right) = n_j^i,$$

which together with the discretised boundary equation forms an algebraic systems of $J-1$ equations, and then the quantities n_j^i ($0 \leq i \leq J$) can be solved.

8 Supporting information S8. Equivalence of fitness measures

We prove the equivalence of the following two measures of invasion fitness:

$$R_0(x, x') := \int_0^\infty p(a) \int_a^\infty S_E(x, x', a) f(x, x', a, s(a-t)) S_I(x, x', a, t) S_P(a, t)\ \mathrm{d}t\ \mathrm{d}a, \quad (3)$$

and

$$R_1(x, x') := \frac{I_{x'}}{I_x}, \quad (4)$$

where

$$I_{x'} := \int_0^\infty p(a) \int_{s_b}^\infty f(x, x', a, s) n(x, x', a, s)\ \mathrm{d}s'\ \mathrm{d}a, \quad (5)$$

is the seed rain rate of a mutant strain with trait value x' in the environment set by the resident species with trait value x. Here, $p(a)$ is the frequency of patches that have existed for a time a, $S_E(x, x', a) = \pi_0 \pi_1(x, x', a, s_b)$ is the establishment probability of a mutant seed that enters a patch of age a in the environment set by the resident, $S_I(a, t)$ and $S_P(a, t)$ are the probabilities that the mutant individual and the patch survives from age a to t, respectively. Note that the individual survival is conditioned upon the patch remaining extant. Furthermore, $f(x, x', a, s)$ is the expected instantaneous fecundity rate of a mutant individual with size s in a patch of age ta. The size (mass of leaf or height) is indicated by s and the size at birth is assumed to be s_b. For a single growth trajectory, we write $s = s(t)$ where t is the time since germination; it follows that $s(0) = s_b$. We refer to the main text and in Supporting information S1-S7 for further explanations of the model and the model ingredients.

The first measure (3) is the basic reproduction ratio of the mutant (Falster et al., 2017). If this is greater than one, a mutant can invade, and vice versa. This measure is

convenient when the characteristic method is employed to solve the master PDE equation, but inconvenient when the upwind method is employed. In the latter situation, the second measure (4) is more efficient since $n(x, x', a, s)$ is known for all ages and sizes, not only for the caracteristc curves. We below show that these two measures are actually equivalent. To this aim, we first establish two identities in Section 1 and 2. The proof of equivalence is then presented in Section 3.

8.1 Patch frequency distribution

The frequency-density of patches with age a is based on the Von Foerster - McKendrick equation at equilibrium. At equilibrium, this reduces to

$$\frac{\partial}{\partial a} p(a) = -\gamma(a) p(a),$$

with the boundary condition

$$p(0) = \int_0^\infty \gamma(a) p(a)\ \mathrm{d}a,$$

which has the solution

$$p(a) = p(0) \exp\left(-\int_0^a \gamma(\tau)\ \mathrm{d}\tau\right),$$

where $\gamma(a)$ is the age-dependent mortality rate of a patch. It is easy to see that $p(a) = p(t) S_P(t, a)$ holds for any $0 \leq t \leq a$, where

$$S_P(t, a) = \exp\left(-\int_t^a \gamma(\tau) d\tau\right) \tag{6}$$

is the survival probability of the patch from the time t to a.

8.2 Seed rain

Here we will show that

$$\int_{s_b}^{s(a)} n(x, x', a, s) f(x, x', a, s)\, ds = \int_0^a I_x S_E(x, x', t) f(x, x', a, s(a-t)) S_I(x, x', t, a)\ \mathrm{d}t. \tag{7}$$

The left-hand side of (7) gives rise to the total seed rain of the mutant population at age a, which is evaluated by integrating population density and fecundity at age a over all attainable size s. The right-hand has the same meaning but is is evaluated as the cumulative contribution of all individuals starting to grow at time $t \leq a$.

At age a, the size of the mutant population ranges from s_b to $s(a)$. The maximum size corresponds to the cohort entering the community at time $a = 0$. For any given size $s \in [s_b, s(a)]$, the entering time $t = t(s) \leq a$ is uniquely determined from the associated characteristic curve defined by

$$\frac{d\hat{s}}{d\tau} = g(x, x', \hat{s}, a), \text{with} \quad \hat{s}(t) = s_b \text{ and } \hat{s}(a) = s. \tag{8}$$

Since the influx rate of the mutant population is constant $n(x, x', s_b, t)g(x, x', s_b, t) = I_x$, we can find the growth flux at age a and size s

$$n(x, x', a, s)g(x, x', a, s) = I_x S_E(x, x', t) S_I(x, x', a, t). \tag{9}$$

Finally, we have

$$\begin{aligned}
& \int_0^a I_x S_E(x, x', t) f(x, x', a, s(a-t)) S_I(x, x', a, st)\ \mathrm{d}t \\
= & \int_{s_b}^{s(a)} n(x, x', a, s) g(x, x', a, s) f(x, x', a, s) \frac{\mathrm{d}s}{g(x, x', a, s)} \\
= & \int_{s_b}^{s(a)} n(x, x', a, s) f(x, x', a, s)\ \mathrm{d}s.
\end{aligned}$$

8.3 Proof of equivalence in measure

Using Eq. (7), the second measure (4) can be rewritten as

$$\begin{aligned}
r(x, x') &= \frac{1}{I_x} \int_0^\infty S_E(x, x', a) p(a) \int_{s_b}^\infty f(x, x', a, s) n(x, x', a, s)\ \mathrm{d}s\ \mathrm{d}a \\
&= \frac{1}{I_x} \int_0^\infty S_E(x, x', a) p(a) \int_{s_b}^{s(a)} f(x, x', a, s) n(x, x', a, s)\ \mathrm{d}s\ \mathrm{d}a \\
&= \int_0^\infty p(a) \int_0^a S_E(x, x', t) f(x, x', a, s(t-a)) S_I(x, x', t, a)\ \mathrm{d}t\ \mathrm{d}a.
\end{aligned}$$

For convenience, denote $f(x, x', a, s(t-a))S_I(x, x', t, a)$ by $F(x, x', t, a)$. We have

$$r(x, x') = \int_0^\infty p(a) \int_0^a S_E(x, x', t) F(x, x', t, a)\ \mathrm{d}t\ \mathrm{d}a.$$

Introduce the indicator function $\xi_{[0,a]}(t)$ which is one if $0 < t < a$ and zero otherwise. We can write

$$r(x, x') = \int_0^\infty \int_0^\infty p(a) \xi_{[0,a]}(t) S_E(x, x', t) F(x, x', t, a)\ \mathrm{d}t\ \mathrm{d}a.$$

Swap t and a without making any other change to the integral,

$$r(x, x') = \int_0^\infty \int_0^\infty p(t) \xi_{[0,t]}(a) S_E(x, x', a) F(x, x', a, t)\ \mathrm{d}a\ \mathrm{d}t.$$

Change the order of integration

$$r(x, x') = \int_0^\infty \int_0^\infty p(t) \xi_{[0,t]}(a) S_E(x, x', a) F(x, x', a, t)\ \mathrm{d}t\ \mathrm{d}a.$$

Note that, as a function of t, $\xi_{[0,t]}(a)$ is 0 for $t < a$ and 1 for $t > a$. Hence $\xi_{[0,t]}(a) = \xi_{[a,\infty]}(t)$. Using this we have

$$r(x, x') = \int_0^\infty \int_0^\infty p(t)\xi_{[a,\infty]}(t) S_E(x, x', a) F(x, x', a, t) \text{ d}t \text{ d}a,$$

which can be rewritten as

$$r(x, x') = \int_0^\infty \int_a^\infty p(t) S_E(x, x', a) F(x, x', a, t) \text{ d}t \text{ d}a,$$

Finally, $p(t) = p(a) S_P(a, t)$ and get

$$r(x, x') = \int_0^\infty p(a) \int_a^\infty S_P(a, t) S_E(x, x', a) F(x, x', a, t) \text{ d}t \text{ d}a.$$

Hence, $R_1(x, x') = R_0(x, x')$ and the two fitness-measures are thus identical.

8.4 Reference

Falster, D. S., Brännström, Å., Westoby, M., & Dieckmann, U. (2017). Multitrait successional forest dynamics enable diverse competitive coexistence. Proceedings of the National Academy of Sciences, 114(13), E2719-E2728.

9 Supporting information S9. Fitness landscape

We find that the resident strategy is always evolutionarily stable. At the evolutionarily stable strategy (ESS) the resident strategy (green dot) is at the fitness maximum, which means that all surrounding mutant strategies will have a negative invasion fitness, as shown in Fig. S1.

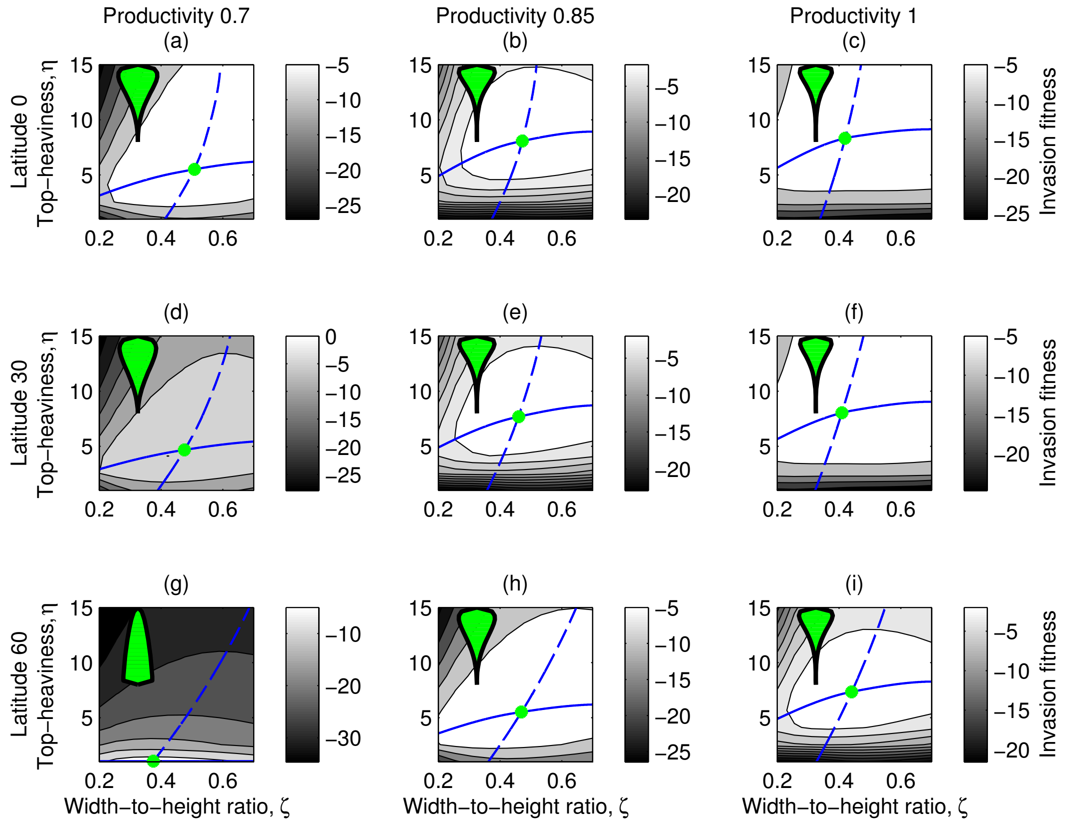

Figure S2. The mutant invasion fitness for the resident ESS at the crossing of the nullclines in Fig. 3.
